# Data-driven space planning: using Suma to collect data

**DOI:** 10.5195/jmla.2019.757

**Published:** 2019-10-01

**Authors:** Erin R. B. Eldermire

**Affiliations:** Head, Flower-Sprecher Veterinary Library, Cornell University Library, Veterinary College, Cornell University, Ithaca, NY, erb29@cornell.edu

## Abstract

Library users frequently make individual requests to staff about how they would like us to improve the services and resources, but it can be difficult to prioritize such requests. To proactively understand how we can improve our library, library staff undertook a comprehensive assessment of spaces and resources using Suma.

At the Flower-Sprecher Veterinary Library (FSVL), users frequently make individual requests to staff about how they would like us to improve the services and resources that we provide. Although it is helpful to receive such requests, it can be difficult to prioritize which to fulfill and which to defer, particularly in light of the limited budget and staff resources that we have. To proactively understand how we can improve our library, staff undertook a comprehensive assessment of our spaces and resources.

The FSVL is a small academic veterinary branch library at Cornell University, which includes 121 seats, gets approximately 90,000 annual visits, and is staffed by a combination of 5 staff members (who together comprise 3.75 full time employees) and 10 student workers.

In the fall of 2018, we used Suma to collect information on how our library users are utilizing our furniture, resources, and spaces. Suma is open-source, tablet-based software for collecting and analyzing observational data to help understand space and resource use. Users define the spaces that they want to assess with an intuitive interface to capture observational data (Figure 1). We defined seven spaces for the FSVL. Data are stored in the cloud and can be analyzed either with built-in tools or can be exported for analysis outside of Suma.

**Figure 1:**
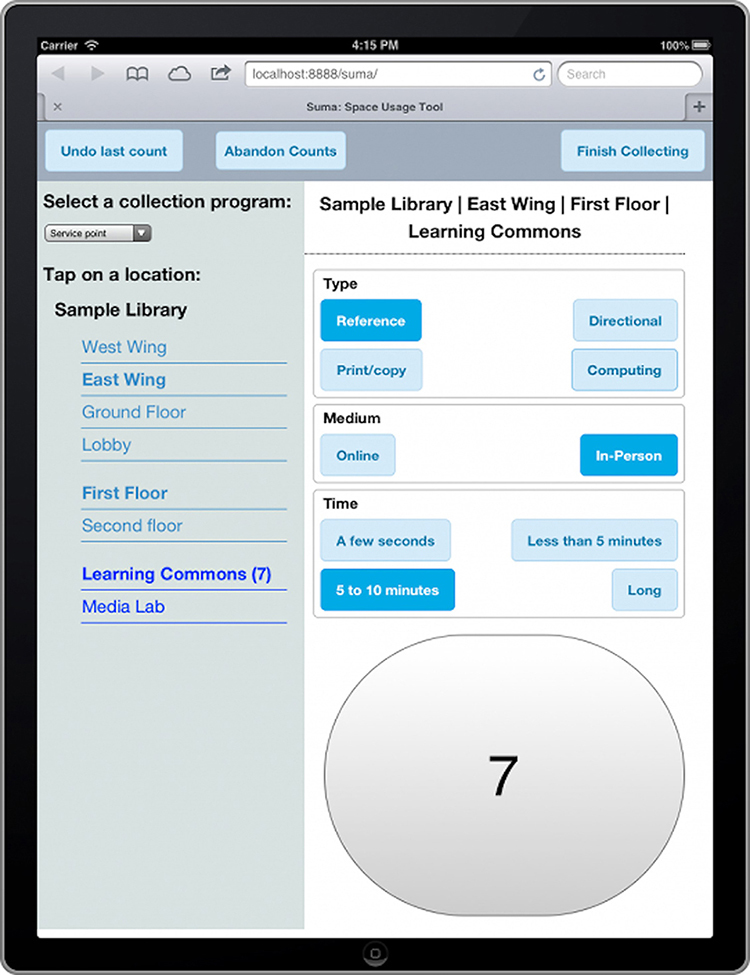
Suma data collection example screen

We started with a five-day testing period, during which we tested Suma, simulated data collection, and defined the spaces and resources that we wished to target. Once we were ready to collect data, we trained all participating student workers and staff in using Suma and ensured consistency between data collectors by clearly defining parameters.

We collected data for nine weeks (of an eighteen-week semester) that were targeted to capture the variety of library uses over the semester, including the semester start, mid-semester, and semester end dates. Data were collected weekdays at five time periods and weekends at two time periods. These time periods were chosen to capture uses that varied throughout the day, balanced with student and staff availability. Data collection took five to fifteen minutes per session. Three staff members and four student workers were involved in collecting data.

Suma was easy to use, and trends quickly emerged to help us make informed decisions, streamline our efforts and resources, and uncover future assessment needs. Using Suma on a tablet allowed us to easily take photos with the built-in camera. Like any observational data, however, sampling times can bias the outcomes. To address potential variation between data collectors, we defined the parameters of the project in a document that all of us were given, and each data collector did a trial run with the project lead to address questions and to norm data collection methods.

Using Suma helped us to come to valuable conclusions. For instance, we documented low use of our public desktop computers and frequent use of personal laptops, so we replaced only half of our desktops but added monitors with docking stations for library users to connect their laptops to. We learned that our carrels are wildly popular and that chairs without a table or desk are hardly used. We also discovered that individuals use many electronic devices concurrently (e.g., laptop, phone, and tablet) and that electrical plugs are in high demand, so we installed additional plugs and surge strips throughout the library.

To further understand our results, we conducted additional assessments, such as a paper survey to understand why the carrels were popular and focus groups to understand pain points and gather recommendations from our users. Ultimately, using Suma helped us to capture basic use patterns to inform near- and long-term plans to improve our space and services.

**Erin R. B. Eldermire, MLS**, erb29@cornell.edu, http://orcid.org/0000-0001-5846-40, Head, Flower-Sprecher Veterinary Library, Cornell University Library, Veterinary College, Cornell University, Ithaca, NY

